# *Impatiens pandurata* (Balsaminaceae), a new species from Yunnan, China

**DOI:** 10.1186/s40529-015-0108-4

**Published:** 2015-10-23

**Authors:** Yun-Hong Tan, Yan-Nan Liu, Hong Jiang, Xin-Xin Zhu, Wei Zhang, Sheng-Xiang Yu

**Affiliations:** 1 Center for Integrative Conservation, Xishuangbanna Tropical Botanical Garden, Chinese Academy of Sciences, Menglun, Mengla, 666303 Yunnan China; 2 State Key Laboratory of Systematic and Evolutionary Botany, Institute of Botany, Chinese Academy of Sciences, Beijing, 100093 People’s Republic of China; 3 Yunnan Academy of Forestry/Yunnan Laboratory for Conservation of Rare, Endangered and Endemic Forest Plants, State Forestry Administration, Kunming, 650201 China; 4 Shanghai Chenshan Plant Science Research Center, Chinese Academy of Sciences, Shanghai Chenshan Botanical Garden, Shanghai, 201602 China; 5 Key Laboratory of Economic Plants and Biotechnology, Kunming Institute of Botany, Chinese Academy of Sciences, Kunming, 650201 Yunnan China

**Keywords:** Balsaminaceae, *Impatiens*, Morphology, New species, Phylogeny

## Abstract

**Background:**

The species-rich genus *Impatiens* is mainly distributed throughout much of tropical Africa, India, southwest Asia, southern China and Japan. There are more than 270 species recorded in China, most of which are restricted to the southwest. An unknown species of *Impatiens* was collected from Yunnan, southwest China.

**Results:**

*Impatiens pandurata* Y. H. Tan & S. X. Yu, a new species of Balsaminaceae from Jinping County and Malipo County, Yunnan, China is similar to *I. apalophylla* and *I. clavigera* in having racemose inflorescences, 4 lateral sepals, hammer-shaped capsules and ellipsoid seeds, but differs in having leaves with oblanceolate blades aggregated at the top of the stem, 3–5-flowered racemes, a yellow lower sepal without reddish patches, yellowish flowers, and a dorsal petal with stalks at the base. Molecular phylogenetic analyses of sequences from both nuclear ribosomal and plastid genes confirm that this new species is distinct from morphologically similar species previously recorded.

**Conclusion:**

With the support of careful morphological studies and phylogenetic analysis, *I. pandurata* is a species new to science.

**Electronic supplementary material:**

The online version of this article (doi:10.1186/s40529-015-0108-4) contains supplementary material, which is available to authorized users.

## Background

The genus *Impatiens* L. (Balsaminaceae), containing over 1000 species (Grey-Wilson 1980; Fischer 2004; Yu et al. 2015), is mainly distributed throughout much of tropical Africa, India, South-west Asia, southern China and Japan, with only a few species spreading into the north temperate zone of Europe, Russia and China as well as North America (Grey-Wilson 1980). *Impatiens* species occur in diverse habitats, from sea level to 4000 m in elevation, in forest understories, roadside ditches, valleys, abandoned fields, along streams and in seepage, usually in mesic or wet conditions, although some species can tolerate drier habitats (Yu et al. 2015). Because of its species diversity, the genus has been regarded as ‘the dicot counterpart of the orchid’ (Yuan et al. 2004).

Five diversity hotspots for *Impatiens* have been recognized, i.e. tropical Africa, Madagascar, southern India and Sri Lanka, the eastern Himalayas, and southeast Asia (Song et al. 2003; Yuan et al. 2004). *Impatiens* is notoriously difficult to classify morphologically (Hooker 1908; Grey-Wilson 1980) and the semi-succulent stems, fleshy leaves, and extremely fragile flowers make it challenging to prepare well-dried herbarium specimens. The publication of new species each year shows that the genus has been under-collected and under-studied (e.g. Narayanan et al. 2013; Utami 2013; Kuang et al. 2014; Luo et al. 2014).

There are more than 270 species of *Impatiens* recorded in China (Yu 2012; Chen 2001; Chen et al. 2007), most of them restricted to the southwest. During recent field expeditions in Yunnan, the authors collected several specimens with distinctive morphological characteristics. After careful consultation of the literature and specimens, we concluded that these specimens are morphologically distinct from any described species. After additional molecular phylogenetic analysis, we are confident that this species is new to science.

## Methods

### Morphology

Characteristics of the leaves, inflorescence and flowers were described and measured on both dried herbarium specimens (from HITBC and PE) and fresh specimens in the field.

### Molecular methods

DNA sequences of 151 species of *Impatiens* were used and three species, *Hydrocera triflora* (L.) Wight & Arn. (Balsaminaceae), and *Marcgravia umbellata* L. and *Norantea* *guianensis* Aubl. (Marcgraviaceae), were included as outgroups, based on the results of Yuan et al. (2004), Janssens et al. (2006) and Yu et al. (2015). All sequences were downloaded from GenBank, except those of the new species, *I. pandurata*, which were newly generated for this study (Genbank accession numbers XXXX, XXXX for ITS, XXXX, XXXX for *atpB*-*rbcL* and XXXX, XXXX for *trnL*-*F*). Vouchers and GenBank accession numbers are listed in Additional file 1: Table S1.

Three molecular markers were used: ITS, *atpB*-*rbcL* and *trnL*-*F*. Total genomic DNA was extracted from silica gel-dried leaves using a modified CTAB protocol from Doyle and Doyle (1987). Primers and PCR protocols for ITS, *atpB*-*rbcL* and *trnL*-*F* are derived from White et al. (1990), Janssens et al. (2006) and Taberlet et al. (1991), respectively. PCR products were purified using a GFX™PCR DNA and Gel Band Purification Kit (Amersham Pharmacia Biotech, Piscataway, NJ, USA). Sequencing reactions were carried out using an ABI Prism Bigdye Terminator Cycle Sequencing Kit (Applied Biosystems, Foster City, CA, USA). Products were analyzed on an ABI3730xl automated DNA sequencer. Sequences were aligned using the default parameters in Clustal X v.1.83 (Thompson et al. 1997) and further adjusted manually in BioEdit v.7.0 (Hall 1999). Four difficult-to-align regions in *trnL*-*F* (encompassing 73 sites) and one difficult-to-align region in *atpB*-*rbcL* (encompassing 42 sites) were excluded from the analyses.

Maximum parsimony (MP) and Bayesian inference (BI) were used to analyze the ITS and plastid data sets. The MP analyses were carried out in PAUP* v.4.0b10 (Swofford 2003). Heuristic searches were conducted with 1000 replicates of random addition, one tree held at each step during stepwise addition, tree-bisection-reconnection (TBR) branch swapping, MulTrees in effect, and steepest descent off. Bootstrapping was conducted with 1000 replicates with 10 random taxon additions and heuristic research options. The BI analyses were carried out in MrBayes v.3.0b4 (Ronquist and Huelsenbeck 2003). Each of the three regions (ITS, *atpB*-*rbcL*, and *trnL*-*F*) was assigned its own model of nucleotide substitution, as determined by the Akaike information criterion (AIC) in Modeltest v.3.06 (Posada and Crandall 1998).

## Results and discussion

### *Impatiens pandurata* Y. H. Tan & S. X. Yu, sp. nov.

This species is similar to *I. apalophylla* and *I. clavigera* in having racemose inflorescences, 4 lateral sepals, hammer-shaped capsules and ellipsoid seeds, but differs in having leaves with oblanceolate blades aggregated at the top of the stem, 3–5-flowered racemes, a yellow lower sepal without reddish patches, yellowish flowers, and a dorsal petal with stalks at the base.

Type: CHINA. Yunnan, Malipo County, Tianbao, Bajiaoping, limestone forests, elev. 1250 m, 22°57′44″ N, 104°51′45″ E, 30 Oct 2012, *Yun*-*Hong Tan* *5728* (holotype: HITBC; isotype: PE). Paratype: China. Yunnan: Malipo County, Tianbao Town, elev. 1200 m, 23° 01′02.47″ N, 104°49′34.19″ E, 20 Nov 2014, *Xin*–*Xin Zhu 0001* (CSH)

 Figs. 1, 2.

**Fig. 1:**
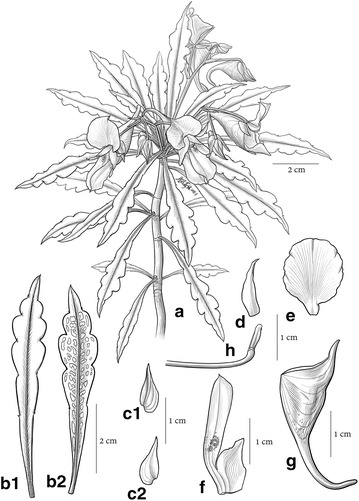
*Impatiens pandurata* Y. H. Tan & S. X. Yu. **a** Habit;** b**, ** b**_**1**_,** b**_**2**_ Leaf, adaxial surface and abaxial surface;** c**,** c**_**1**_,** c**_**2**_ Outer lateral sepal, abaxial surface and adaxial surface; **d** Inner lateral sepal; **e** dorsal petal; **f** lateral united petal; **g** lower sepal, lateral view; **h** capsule, immature. All from *Tan 5728* (HITBC) and drawn by Yun-Xi Zhu

**Fig. 2:**
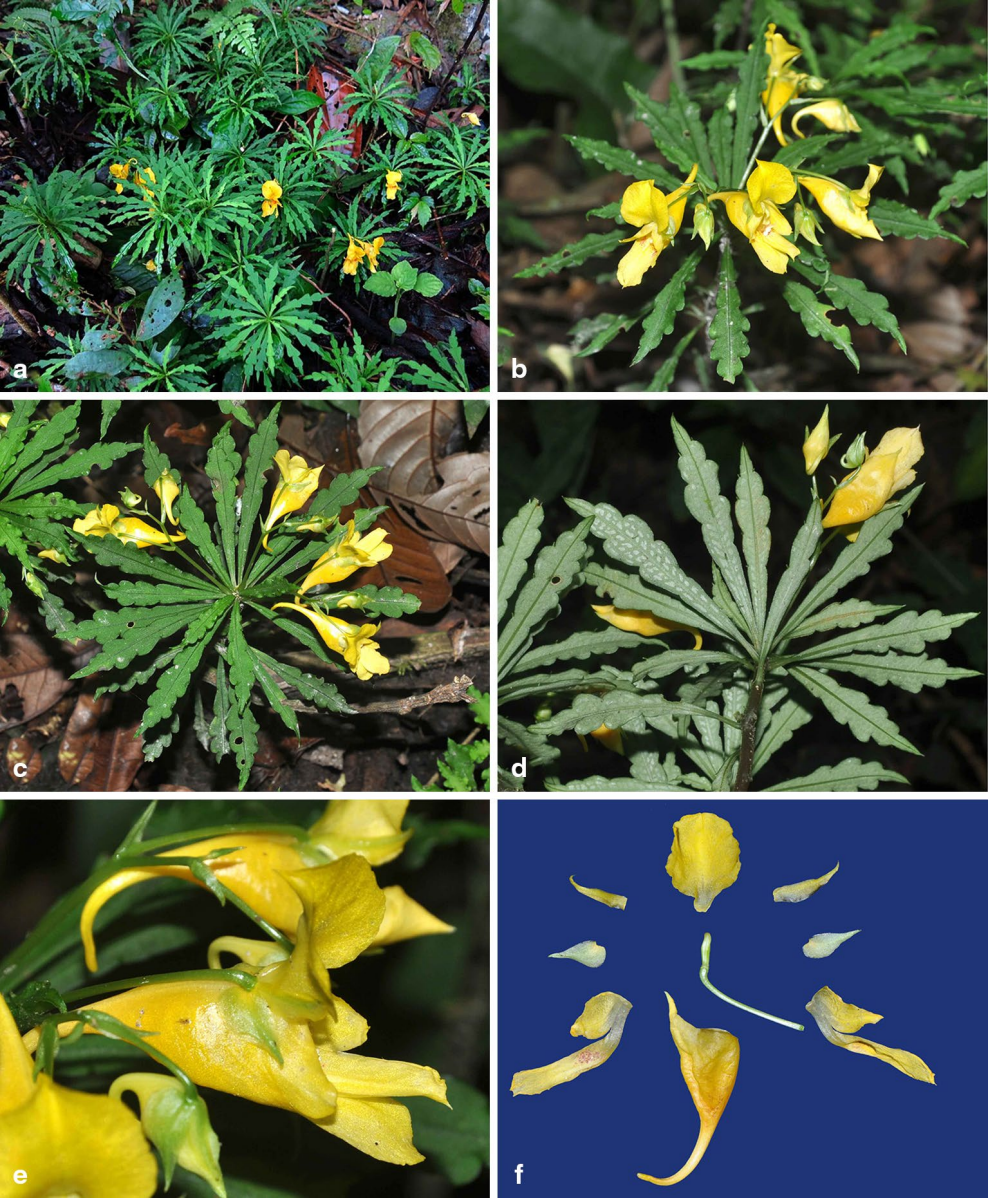
*Impatiens pandurata* Y. H. Tan & S. X. Yu. **a** Habitat; **b** habit; **c** leaf adaxial surface; **d** leaf abaxial surface; **e** flower, lateral view; **f** flower, different parts in separation, front view. All from *Tan 5728* (HITBC)

Herb perennial, 20–30 cm tall, glabrous. Stems fleshy, erect, simple or branched; inferior nodes unapparent. Leaves alternate, aggregated at stem apex, blades oblanceolate to linear oblanceolate, 5–7 cm long, 1–1.5 cm wide, apex acuminate, base cuneate, deep green above, pale green beneath, sometime with grey patches, margin deeply crenate, with spinose teeth. Veins unapparent. Petioles 0.8–1.2 cm. Racemes solitary in the upper axils, 5–7 cm long, 2–3 (−5)-flowered. Pedicels thin, 15–20 mm long. Bracts ovate to lanceolate, 7–9 mm long, acute. Flowers yellowish or cream. Lateral sepals 4, the outer 2 large, ovate to lanceolate, inaequilateral, 2–3 veined, yellowish-green, base rounded, apex acuminate to caudate, 7.8–9.1 × 3.3–3.7 mm; the inner 2 small, 10.7–11.4 × 1.2–1.6 mm, inaequilateral, apex acuminate. Lower sepal 2.5–3.0 cm long excluding spur, saccate, spur 5–5.6 mm. Dorsal petal 12.5–13.5 mm long, 11.6–12.3 mm wide, orbicular, apex rounded, base broadly cuneate and abruptly constricted into a stalk, midrib obvious, with a slight dorsal crest. Lateral united petals 2.1–2.4 cm long, the lower lobes 11.5–12.5 mm long, 5–5.5 mm wide, oblong, the upper ones 21–24 mm long, 4.5–5.5 wide, elliptic, apex emarginate, middle of inner margin without appendage. Stamens 5, filaments linear, 2–3 mm long, anthers obtuse. Ovary clavate, superior part inflated. Capsule hammer-shaped, seed ellipsoid.

*Phenology* Flowering and fruiting from September to December.

*Ecology* This new species grows under evergreen broad leaf forest; 1000–1200 m.

*Distribution* *Impatiens pandurata* is known from Jinping County and Malipo County, Yunnan, China.

*Etymology* The specific epithet ‘pandurata’ refers to the leaf shape of the new species.

## Discussion

**Fig. 3:**
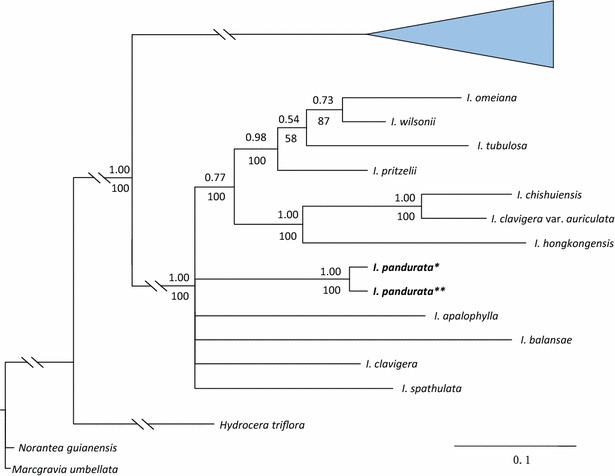
Partial Bayesian consensus phylogram based on the branch lengths of the ITS data. *Numbers* above and below branches are Bayesian posterior probabilities (>0.5) and bootstrap percentages (>50 %), respectively. “−” indicates nodes not supported

**Fig. 4:**
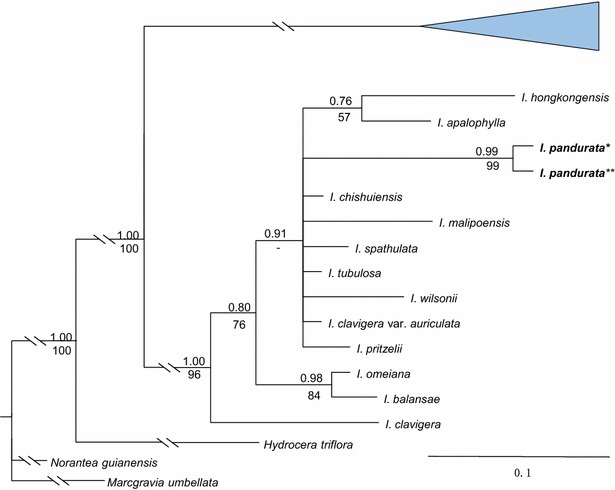
Partial Bayesian consensus phylogram based on the branch lengths of the cpDNA data (*atpB*-*rbcL* + *trn L*-*F*). *Numbers* above and below branches are Bayesian posterior probabilities (>0.5) and bootstrap percentages (>50 %), respectively. “−” indicates nodes not supported

The phylogenetic topologies obtained with ITS and *atpB*-*rbcL* + *trnL*-*F* are congruent with those of previous studies (Yu et al. 2015). Both ITS and *atpB*-*rbcL* + *trnL*-*F* indicate that *I. pandurata* is a distinct member of the basal clade, subgenus *Clavicarpa* Yu et al. (2015) (Figs. 3, 4, Additional file 2: Figure S1, Additional file 3: Figure S2). The morphological characters, including perennial herb, racemose inflorescence, 4 lateral united petals, 4-carpellate ovary and one ovule per carpel, also support membership of *I. pandurata* in this subgenus. Although the ITS data shows that *I. pandurata* belongs to the basal clade, the relationships among the species in this clade are unclear. In the *atpB*-*rbcL* + *trnL*-*F* tree, *I. pandurata* and other species form a large polytomy, so the relationships among subgenus *Clavicarpa* are also unresolved. However, both nuclear ribosomal and plastid genes agree with the morphological evidence that *I. pandurata* is a new and distinct species.

The diagnostic morphological characters that distinguish *I. pandurata* from its allies are the oblanceolate leaves aggregated on the stem apex, with white macula beneath, the narrow lanceolate bracteole, and the subrotund dorsal petal with apparent stalk. Four species with similar morphological characters, *I. pandurata*, *I. apalophylla*, *I. clavigera*, and *I. spathulata*, are compared with each other, on the basis of their reproductive and vegetative characters in Table 1.

**Table 1:** Comparison among *Impatiens pandurata*, *I. apalophylla, I. clavigera* and *I. spathulata*

Characters	*I. pandurata*	*I. apalophylla*	*I. clavigera*	*I. spathulata*
Plant	Glabrous	Glabrous	Glabrous	Puberulous
Shape of leaf	Oblanceolate	Ovate to oblanceolate	Obovate to oblanceolate	Obovate
Size of leaf	5–7 cm × 1–1.5 mm	10–22 cm × 4–8 cm	5–15 cm × 3–5 cm	6–11 cm × 2.5–3.5 cm
Inflorescence	3–5-flowered	4–10-flowered	5–9-flowered	2–4-flowered
Floral color	Yellow	Yellow with reddish patches	Yellow	Pink
Outer lateral sepals	Oblique, ovate-orbiculate, aequilateral, apex acuminate	Oblique ovate, apex acuminate	Oblique ovate, apex acuminate	Oblique ovate to ovate, apex acuminate
Inner lateral sepals	Linear	Lanceolate	Linear-lanceolate	Linear-lanceolate
Lower sepal	Absent patch	With redish patches	Absent patch	Absent patch
Spur	Long, 1–1.5 cm	Long, 1.5–2 cm	Short, 5–6 mm	Long, 1.5–1.8 cm
Lower lobe of united petals	8–12 mm long, elliptic	1–1.5 cm long, oblong	1–1.2 cm long, oblong	1–1.2 cm long, oblong
Upper lobe of united petal	2–2.5 cm long, oblong, yellow, with slightly reddish patches	2.5–2.7 cm long, oblong, yellow, with apparent reddish patches	2.5–2.6 cm long, oblong, yellowish-green, absent patch	2.3–2.5 cm long, oblong, pink, absent patch
Dorsal petal	Suborbicular	Elliptic	Obovate	Elliptic to ovate

## Conclusion

With the support of morphological studies and molecular phylogenetic analysis, *I. pandurata* is a species new to science. Detailed descriptions, line drawings, color plates, phylogenetic analysis and comparisons with phenetically similar species are provided to aid in identification.

## Additional files

**Additional file 1: Table S1.** Species, GenBank accession numbers and vouchers for the sequences used in this study.

**Additional file 2: Figure S1.** Bayesian consensus phylogram based on the branch length of the ITS data. Numbers above and below branches are Bayesian posterior probabilities (> 0.5) and bootstrap percentages (> 50%), respectively. “-” indicates nodes not supported.

**Additional file 3: Figure S2.** Bayesian consensus phylogram based on the branch length of the cpDNA data (*atpB*-*rbcL* + *trnL*-*F*). Numbers above and below branches are Bayesian posterior probabilities (> 0.5) and bootstrap percentages (> 50%), respectively. “-” indicates nodes not supported.
