# Supplementary figures and images for: Impatiens pandurata (Balsaminaceae), a new species from Yunnan, China
Source: Bot Stud. 2015 Oct 23;56:29. doi: 10.1186/s40529-015-0108-4 (PMC5432899; doi:10.1186/s40529-015-0108-4)

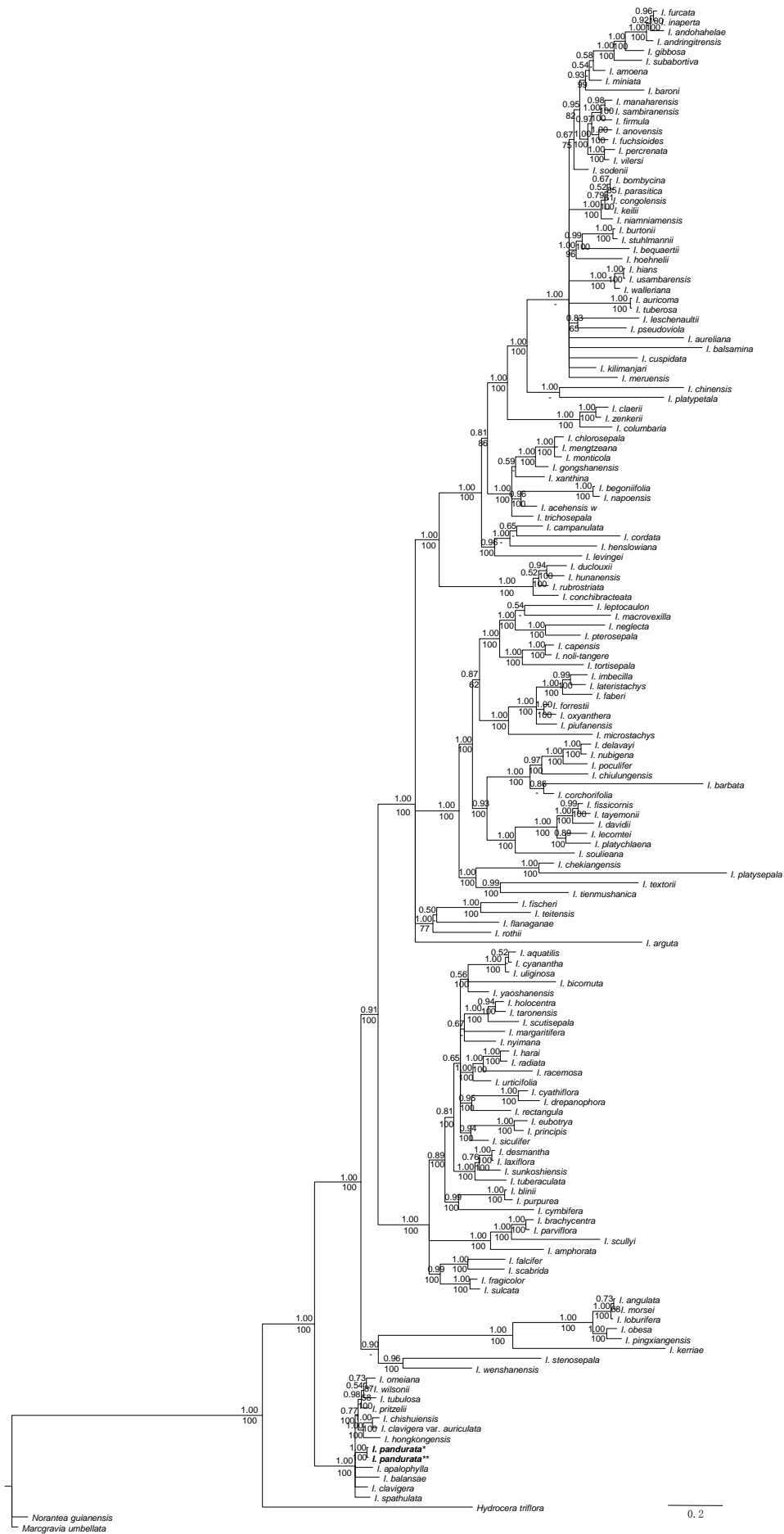

Supplement: Supplementary file 2 — Additional file 2: Figure S1. Bayesian consensus phylogram based on the branch length of the ITS data. Numbers above and below branches are Bayesian posterior probabilities (> 0.5) and bootstrap percentages (> 50%), respectively. “-” indicates nodes not supported. [file 40529_2015_108_MOESM2_ESM.pdf]

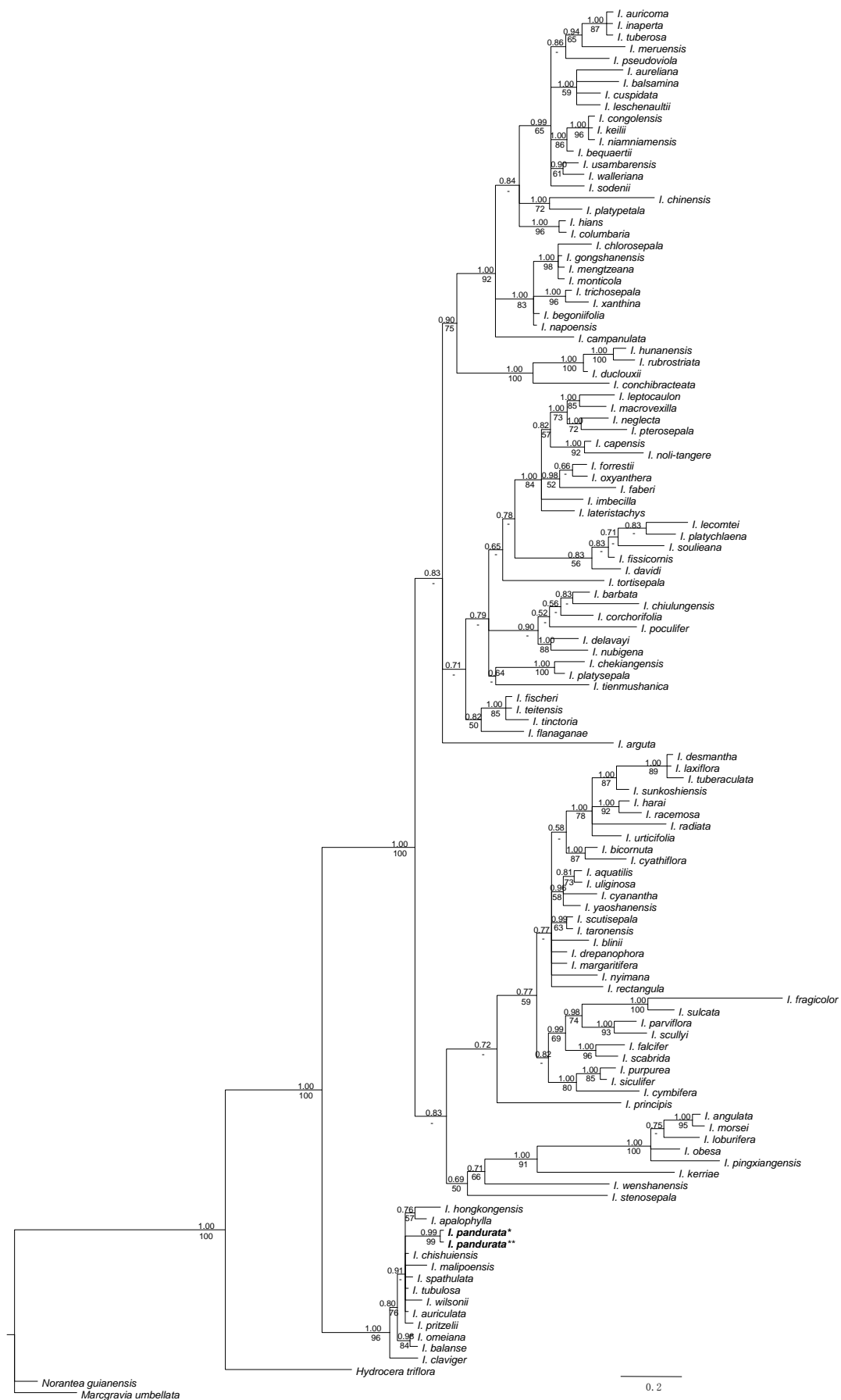

Supplement: Supplementary file 3 — Additional file 3: Figure S2. Bayesian consensus phylogram based on the branch length of the cpDNA data (atpB-rbcL + trnL-F). Numbers above and below branches are Bayesian posterior probabilities (> 0.5) and bootstrap percentages (> 50%), respectively. “-” indicates nodes not supported. [file 40529_2015_108_MOESM3_ESM.pdf]
